# 2-(4-Methyl­phen­yl)-2-oxoethyl 3-bromo­benzoate

**DOI:** 10.1107/S1600536812046995

**Published:** 2012-11-28

**Authors:** Imtiaz Khan, Aliya Ibrar, Artur Korzański, Maciej Kubicki

**Affiliations:** a Department of Chemistry, Quaid-i-Azam University, Islamabad 45320, Pakistan; b Department of Chemistry, Adam Mickiewicz University, Grunwaldzka 6, 60-780 Poznań, Poland

## Abstract

The mol­ecule of the title compound, C_16_H_13_BrO_3_, is built of two approximately planar fragments, *viz.* 3-bromo­benzoate [maximum deviation = 0.055 (2) Å and 2-oxo-2-*p*-tolyl­ethyl [maximum deviation = 0.042 (2) Å], inclined by 46.51 (7)°. In the crystal, weak C—H⋯O hydrogen bonds and Br⋯Br contacts [3.6491 (7) Å] connect the mol­ecules into infinite layers parallel to (-221).

## Related literature

For the structures of similar compounds, see: Fun, Arshad *et al.* (2011 ▶); Fun, Loh *et al.* (2011 ▶); Fun, Ooi *et al.* (2011 ▶); Fun, Shahani *et al.* (2011 ▶).
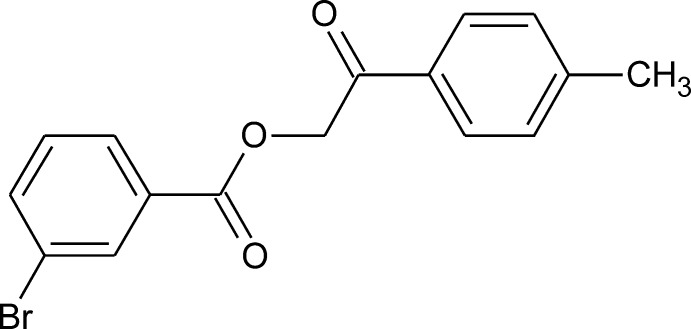


## Experimental

### 

#### Crystal data

C_16_H_13_BrO_3_
*M* *_r_* = 333.17
Triclinic, 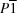

*a* = 4.7977 (3) Å
*b* = 10.9951 (7) Å
*c* = 14.1645 (8) Å
α = 74.829 (5)°
β = 87.758 (5)°
γ = 79.327 (5)°
*V* = 708.64 (7) Å^3^
*Z* = 2
Mo *K*α radiation
μ = 2.90 mm^−1^
*T* = 295 K
0.25 × 0.2 × 0.08 mm

#### Data collection

Agilent Xcalibur Eos diffractometer
Absorption correction: multi-scan (*CrysAlis PRO*; Agilent, 2010 ▶) *T*_min_ = 0.335, *T*_max_ = 1.000
7924 measured reflections
2501 independent reflections
1768 reflections with *I* > 2σ(*I*)
*R*_int_ = 0.026

#### Refinement

*R*[*F* ^2^ > 2σ(*F* ^2^)] = 0.038
*wR*(*F* ^2^) = 0.106
*S* = 1.05
2501 reflections
192 parameters
H-atom parameters constrained
Δρ_max_ = 0.44 e Å^−3^
Δρ_min_ = −0.42 e Å^−3^

### 

Data collection: *CrysAlis PRO* (Agilent, 2010 ▶); cell refinement: *CrysAlis PRO*; data reduction: *CrysAlis PRO*; program(s) used to solve structure: *SIR92* (Altomare *et al.*, 1993 ▶); program(s) used to refine structure: *SHELXL97* (Sheldrick, 2008 ▶); molecular graphics: *SHELXTL* (Sheldrick, 2008 ▶) and *Mercury* (Macrae *et al.*, 2008 ▶); software used to prepare material for publication: *SHELXL97*.

## Supplementary Material

Crystal structure: contains datablock(s) I, global. DOI: 10.1107/S1600536812046995/ng5304sup1.cif

Structure factors: contains datablock(s) I. DOI: 10.1107/S1600536812046995/ng5304Isup2.hkl

Supplementary material file. DOI: 10.1107/S1600536812046995/ng5304Isup3.cml

Additional supplementary materials:  crystallographic information; 3D view; checkCIF report

## Figures and Tables

**Table 1:** Hydrogen-bond geometry (Å, °)

*D*—H⋯*A*	*D*—H	H⋯*A*	*D*⋯*A*	*D*—H⋯*A*
C5—H5⋯O10^i^	0.93	2.44	3.198 (4)	139
C9—H92⋯O7^ii^	0.97	2.56	3.406 (4)	146

Symmetry codes: (i) 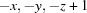; (ii) 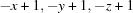.

**Fig. 1.**
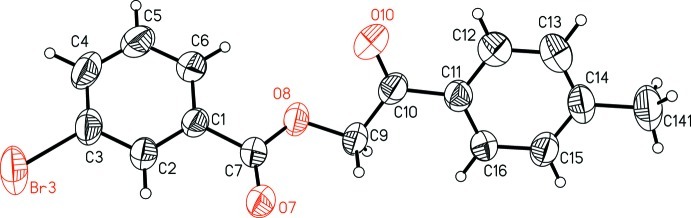
Anisotropic ellipsoid representation of 1 together with atom labelling scheme. The ellipsoids are drawn at 50% probability level, hydrogen atoms are depicted as spheres with arbitrary radii.

**Fig. 2.**
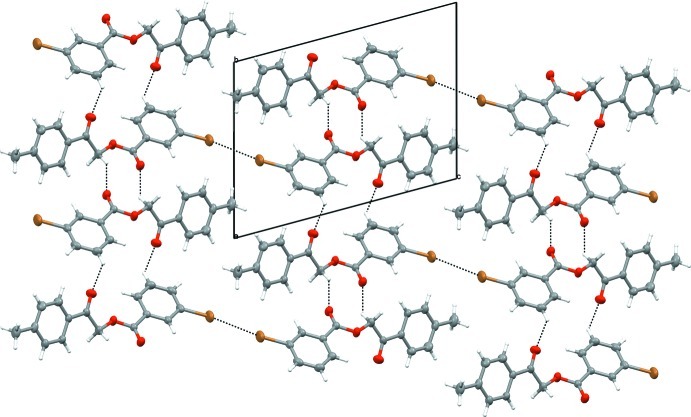
The layer of the molecules connected by weak C—H···O and Br···Br interactions

## supplementary crystallographic information

### Comment

Keto esters, an important class of versatile intermediates, are extensively used in agrochemical, pharmaceutical, and dyestuff industries. They are also useful organic building blocks for the synthesis of complex natural products and are frequently employed synthons in organic synthesis, especially in heterocyclic synthesis. Prompted by literature findings, we herein report the synthesis of 2-(4-methylphenyl)-2-oxoethyl 3-bromobenzoate which can be used as an effective synthon in heterocyclic chemistry. The formation of keto ester (1) was confirmed by the changes in the spectral properties such as IR absorptions, ^1^H and ^13^C NMR signals for dominant functional groups. The conformation of molecule (1) can be described by the dihedral angle between two approximately planar fragments: 3-bromobenzoate (maximum deviation from the least-squares plane is 0.055 (2) Å) and 2-oxo-2-*p*-tolylethyl (0.042 (2) Å). In the crystal, this angle is 46.51 (7) ° (Fig. 1). In similarly substituted (*para-meta*) analogues, this angle was much smaller: in 2-(4-fluorophenyl)-2-oxoethyl 3-(trifluoromethyl)benzoate (Fun, Arshad *et al.*, 2011) this angle is 20.34 (9)°, in 2-(4-chlorophenyl)-2-oxoethyl 3-(trifluoromethyl)benzoate (Fun, Loh *et al.*, 2011) - 15.50 (8)°; on the other hand, this angle was larger in some other similar compounds: 66.66 (8)° in 2-(4-bromophenyl)-2-oxoethyl 2-methylbenzoate (Fun, Ooi *et al.*, 2011) and 80.70 (7)° in 2-(4-bromophenyl)-2-oxoethyl 4-methylbenzoate (Fun, Shahani *et al.*, 2011).

Weak but directional C—H···O hydrogen bonds and C—Br···Br(*-1 - x,1 - y,-z*) halogen interactions (Br···Br 3.6491 (7) Å, C—Br···Br 164.37 (10) °) connect molecules into layers approximately parallel to (-221) plane (Fig. 2); these planes are interacting with one another by means of weak C—H···O contacts and van der Waals interactions.

### Experimental

2-(4-Methylphenyl)-2-oxoethyl 3-bromobenzoate (1) was synthesized by treating 3-bromobenzoic acid (0.01 mol) with the solution of 2-bromo-1-*p*-tolylethanone (0.01 mol) in *N*,*N*-dimethylformamide (DMF) using triethylamine (TEA) as a catalyst at room temperature for 2 h. Yield: 87%; m.p 96–97°C; *R*_f_: 0.27 (n-hexane: ethyl acetate, 9: 1); IR (neat, cm^-1^): 3034 (C*_sp_*_2_-H), 2924, 2853 (C*_sp_*_3_-H), 1728 (C=O_ester_), 1685 (C=O_keto_), 1585, 1561 (C=C), 1230 (C—O); ^1^H NMR (300 MHz, CDCl_3_): δ 8.08–8.04 (m, 1H, Ar—H), 7.88 (d, 2H, *J* = 8.4 Hz, Ar—H), 7.72–7.68 (m, 1H, Ar—H), 7.45–7.36 (m, 2H, Ar—H), 7.35–7.28 (m, 2H, Ar—H), 5.59 (s, 2H, OCH_2_), 2.44 (s, 3H, CH_3_); ^13^C NMR (75 MHz, CDCl_3_): δ 191.33, 165.44, 145.06, 134.42, 133.02, 132.04, 131.61, 131.21, 129.64, 127.94, 127.30, 122.07, 66.65, 21.85.

Crystals were obtained by recrystallization from ethyl acetate.

### Refinement

Hydrogen atoms were placed geometrically and refined as riding model with isotropic thermal parameters.

### Crystal data

**Table:**

C_16_H_13_BrO_3_	*Z* = 2
*M**_r_* = 333.17	*F*(000) = 336
Triclinic, *P*1	*D*_x_ = 1.561 Mg m^−^^3^
Hall symbol: -P 1	Mo *K*α radiation, λ = 0.71073 Å
*a* = 4.7977 (3) Å	Cell parameters from 2186 reflections
*b* = 10.9951 (7) Å	θ = 3.0–29.0°
*c* = 14.1645 (8) Å	µ = 2.90 mm^−^^1^
α = 74.829 (5)°	*T* = 295 K
β = 87.758 (5)°	Plate, colourless
γ = 79.327 (5)°	0.25 × 0.2 × 0.08 mm
*V* = 708.64 (7) Å^3^	

### Data collection

**Table:**

Agilent Xcalibur Eos diffractometer	2501 independent reflections
Radiation source: Enhance (Mo) X-ray Source	1768 reflections with *I* > 2σ(*I*)
Graphite monochromator	*R*_int_ = 0.026
Detector resolution: 16.1544 pixels mm^-1^	θ_max_ = 25.0°, θ_min_ = 3.0°
ω–scan	*h* = −5→5
Absorption correction: multi-scan (*CrysAlis PRO*; Agilent, 2010)	*k* = −12→13
*T*_min_ = 0.335, *T*_max_ = 1.000	*l* = −16→16
7924 measured reflections	

### Refinement

**Table:**

Refinement on *F*^2^	Primary atom site location: structure-invariant direct methods
Least-squares matrix: full	Secondary atom site location: difference Fourier map
*R*[*F*^2^ > 2σ(*F*^2^)] = 0.038	Hydrogen site location: inferred from neighbouring sites
*wR*(*F*^2^) = 0.106	H-atom parameters constrained
*S* = 1.05	*w* = 1/[σ^2^(*F*_o_^2^) + (0.053*P*)^2^ + 0.0904*P*] where *P* = (*F*_o_^2^ + 2*F*_c_^2^)/3
2501 reflections	(Δ/σ)_max_ = 0.001
192 parameters	Δρ_max_ = 0.44 e Å^−^^3^
0 restraints	Δρ_min_ = −0.42 e Å^−^^3^

### Special details

**Table:**

Geometry. All s.u.'s (except the s.u. in the dihedral angle between two l.s. planes) are estimated using the full covariance matrix. The cell s.u.'s are taken into account individually in the estimation of s.u.'s in distances, angles and torsion angles; correlations between s.u.'s in cell parameters are only used when they are defined by crystal symmetry. An approximate (isotropic) treatment of cell s.u.'s is used for estimating s.u.'s involving l.s. planes.
Refinement. Refinement of *F*^2^ against ALL reflections. The weighted *R*-factor *wR* and goodness of fit *S* are based on *F*^2^, conventional *R*-factors *R* are based on *F*, with *F* set to zero for negative *F*^2^. The threshold expression of *F*^2^ > σ(*F*^2^) is used only for calculating *R*-factors(gt) *etc*. and is not relevant to the choice of reflections for refinement. *R*-factors based on *F*^2^ are statistically about twice as large as those based on *F*, and *R*- factors based on ALL data will be even larger.

### Fractional atomic coordinates and isotropic or equivalent isotropic displacement parameters (Å^2^)

**Table:**

	*x*	*y*	*z*	*U*_iso_*/*U*_eq_	
C1	−0.0123 (5)	0.2958 (2)	0.3969 (2)	0.0458 (6)	
C2	−0.0749 (6)	0.3618 (3)	0.3014 (2)	0.0526 (7)	
H2	−0.0098	0.4380	0.2744	0.049 (8)*	
C3	−0.2350 (6)	0.3141 (3)	0.2459 (2)	0.0600 (8)	
Br3	−0.32035 (10)	0.40451 (4)	0.11376 (3)	0.1043 (2)	
C4	−0.3331 (7)	0.2016 (3)	0.2848 (3)	0.0694 (9)	
H4	−0.4406	0.1701	0.2467	0.079 (10)*	
C5	−0.2713 (7)	0.1369 (3)	0.3799 (3)	0.0715 (9)	
H5	−0.3383	0.0612	0.4067	0.088 (12)*	
C6	−0.1099 (6)	0.1829 (3)	0.4368 (2)	0.0576 (7)	
H6	−0.0674	0.1381	0.5015	0.069 (9)*	
C7	0.1605 (6)	0.3501 (3)	0.4556 (2)	0.0473 (7)	
O7	0.2638 (5)	0.4429 (2)	0.42375 (15)	0.0679 (6)	
O8	0.1859 (5)	0.2813 (2)	0.54790 (14)	0.0658 (6)	
C9	0.3630 (7)	0.3139 (3)	0.6129 (2)	0.0609 (8)	
H91	0.2473	0.3539	0.6581	0.081 (11)*	
H92	0.4775	0.3739	0.5760	0.071 (10)*	
C10	0.5507 (6)	0.1933 (3)	0.6681 (2)	0.0524 (7)	
O10	0.5558 (5)	0.0932 (2)	0.64683 (19)	0.0828 (7)	
C11	0.7323 (6)	0.1995 (3)	0.74883 (19)	0.0492 (7)	
C12	0.9153 (7)	0.0901 (3)	0.7958 (2)	0.0662 (8)	
H12	0.9238	0.0154	0.7758	0.084 (11)*	
C13	1.0847 (7)	0.0896 (3)	0.8713 (3)	0.0729 (9)	
H13	1.2053	0.0143	0.9022	0.090 (11)*	
C14	1.0804 (6)	0.1984 (3)	0.9026 (2)	0.0621 (8)	
C141	1.2672 (8)	0.1975 (4)	0.9864 (3)	0.0861 (11)	
H14A	1.2630	0.2839	0.9899	0.129*	
H14B	1.4583	0.1588	0.9760	0.129*	
H14C	1.1988	0.1493	1.0466	0.129*	
C15	0.8999 (7)	0.3084 (3)	0.8553 (2)	0.0625 (8)	
H15	0.8935	0.3831	0.8751	0.087 (12)*	
C16	0.7272 (6)	0.3098 (3)	0.7786 (2)	0.0567 (8)	
H16	0.6078	0.3852	0.7471	0.057 (8)*	

### Atomic displacement parameters (Å^2^)

**Table:**

	*U*^11^	*U*^22^	*U*^33^	*U*^12^	*U*^13^	*U*^23^
C1	0.0446 (15)	0.0450 (15)	0.0505 (16)	−0.0089 (12)	−0.0063 (12)	−0.0156 (12)
C2	0.0567 (17)	0.0521 (17)	0.0504 (16)	−0.0082 (13)	−0.0129 (13)	−0.0148 (13)
C3	0.0624 (19)	0.0616 (19)	0.0565 (18)	0.0044 (15)	−0.0214 (14)	−0.0239 (15)
Br3	0.1370 (4)	0.1121 (4)	0.0632 (3)	−0.0071 (3)	−0.0476 (2)	−0.0254 (2)
C4	0.063 (2)	0.070 (2)	0.086 (2)	−0.0042 (16)	−0.0257 (17)	−0.0422 (19)
C5	0.076 (2)	0.0570 (19)	0.089 (3)	−0.0188 (16)	−0.0179 (19)	−0.0251 (18)
C6	0.0611 (19)	0.0512 (17)	0.0621 (19)	−0.0100 (14)	−0.0132 (14)	−0.0155 (15)
C7	0.0496 (16)	0.0497 (16)	0.0443 (15)	−0.0114 (13)	−0.0086 (12)	−0.0124 (13)
O7	0.0873 (16)	0.0654 (13)	0.0551 (12)	−0.0380 (12)	−0.0191 (11)	−0.0034 (10)
O8	0.0865 (15)	0.0708 (13)	0.0456 (12)	−0.0411 (11)	−0.0199 (10)	−0.0032 (10)
C9	0.077 (2)	0.0640 (18)	0.0477 (17)	−0.0278 (16)	−0.0182 (16)	−0.0116 (15)
C10	0.0626 (18)	0.0583 (18)	0.0428 (15)	−0.0261 (14)	0.0009 (13)	−0.0140 (13)
O10	0.1036 (18)	0.0669 (14)	0.0898 (17)	−0.0245 (13)	−0.0233 (14)	−0.0314 (13)
C11	0.0514 (17)	0.0560 (16)	0.0409 (15)	−0.0174 (13)	−0.0016 (12)	−0.0080 (13)
C12	0.067 (2)	0.0602 (19)	0.071 (2)	−0.0069 (15)	−0.0087 (17)	−0.0184 (16)
C13	0.061 (2)	0.074 (2)	0.075 (2)	0.0002 (17)	−0.0183 (17)	−0.0108 (18)
C14	0.0527 (18)	0.085 (2)	0.0456 (16)	−0.0219 (16)	−0.0094 (13)	−0.0029 (16)
C141	0.072 (2)	0.114 (3)	0.068 (2)	−0.025 (2)	−0.0261 (18)	−0.006 (2)
C15	0.071 (2)	0.071 (2)	0.0503 (17)	−0.0276 (16)	−0.0097 (15)	−0.0121 (15)
C16	0.069 (2)	0.0532 (17)	0.0463 (16)	−0.0164 (14)	−0.0158 (14)	−0.0035 (14)

### Geometric parameters (Å, º)

**Table:**

C1—C2	1.372 (4)	C9—H92	0.9700
C1—C6	1.382 (4)	C10—O10	1.210 (4)
C1—C7	1.494 (4)	C10—C11	1.488 (4)
C2—C3	1.376 (4)	C11—C12	1.378 (4)
C2—H2	0.9300	C11—C16	1.379 (4)
C3—C4	1.376 (5)	C12—C13	1.367 (5)
C3—Br3	1.895 (3)	C12—H12	0.9300
C4—C5	1.364 (5)	C13—C14	1.376 (5)
C4—H4	0.9300	C13—H13	0.9300
C5—C6	1.382 (4)	C14—C15	1.377 (4)
C5—H5	0.9300	C14—C141	1.512 (4)
C6—H6	0.9300	C141—H14A	0.9600
C7—O7	1.191 (3)	C141—H14B	0.9600
C7—O8	1.325 (3)	C141—H14C	0.9600
O8—C9	1.430 (3)	C15—C16	1.387 (4)
C9—C10	1.501 (4)	C15—H15	0.9300
C9—H91	0.9700	C16—H16	0.9300
			
C2—C1—C6	120.2 (3)	O10—C10—C11	120.7 (3)
C2—C1—C7	118.2 (2)	O10—C10—C9	120.7 (3)
C6—C1—C7	121.5 (2)	C11—C10—C9	118.6 (3)
C1—C2—C3	119.4 (3)	C12—C11—C16	118.4 (3)
C1—C2—H2	120.3	C12—C11—C10	118.4 (3)
C3—C2—H2	120.3	C16—C11—C10	123.2 (3)
C4—C3—C2	120.9 (3)	C13—C12—C11	121.0 (3)
C4—C3—Br3	119.6 (2)	C13—C12—H12	119.5
C2—C3—Br3	119.5 (2)	C11—C12—H12	119.5
C5—C4—C3	119.4 (3)	C12—C13—C14	121.4 (3)
C5—C4—H4	120.3	C12—C13—H13	119.3
C3—C4—H4	120.3	C14—C13—H13	119.3
C4—C5—C6	120.6 (3)	C13—C14—C15	117.9 (3)
C4—C5—H5	119.7	C13—C14—C141	121.3 (3)
C6—C5—H5	119.7	C15—C14—C141	120.9 (3)
C1—C6—C5	119.5 (3)	C14—C141—H14A	109.5
C1—C6—H6	120.3	C14—C141—H14B	109.5
C5—C6—H6	120.3	H14A—C141—H14B	109.5
O7—C7—O8	124.1 (3)	C14—C141—H14C	109.5
O7—C7—C1	124.5 (2)	H14A—C141—H14C	109.5
O8—C7—C1	111.3 (2)	H14B—C141—H14C	109.5
C7—O8—C9	118.8 (2)	C14—C15—C16	121.2 (3)
O8—C9—C10	108.3 (2)	C14—C15—H15	119.4
O8—C9—H91	110.0	C16—C15—H15	119.4
C10—C9—H91	110.0	C11—C16—C15	120.1 (3)
O8—C9—H92	110.0	C11—C16—H16	119.9
C10—C9—H92	110.0	C15—C16—H16	119.9
H91—C9—H92	108.4		
			
C6—C1—C2—C3	−0.2 (4)	O8—C9—C10—O10	7.5 (4)
C7—C1—C2—C3	−179.7 (2)	O8—C9—C10—C11	−173.0 (2)
C1—C2—C3—C4	0.1 (4)	O10—C10—C11—C12	2.9 (4)
C1—C2—C3—Br3	−179.7 (2)	C9—C10—C11—C12	−176.6 (3)
C2—C3—C4—C5	0.2 (5)	O10—C10—C11—C16	−177.5 (3)
Br3—C3—C4—C5	−180.0 (2)	C9—C10—C11—C16	3.1 (4)
C3—C4—C5—C6	−0.5 (5)	C16—C11—C12—C13	1.2 (5)
C2—C1—C6—C5	−0.1 (4)	C10—C11—C12—C13	−179.2 (3)
C7—C1—C6—C5	179.4 (3)	C11—C12—C13—C14	−0.5 (5)
C4—C5—C6—C1	0.4 (5)	C12—C13—C14—C15	−0.1 (5)
C2—C1—C7—O7	−5.0 (4)	C12—C13—C14—C141	179.8 (3)
C6—C1—C7—O7	175.5 (3)	C13—C14—C15—C16	0.1 (5)
C2—C1—C7—O8	175.4 (2)	C141—C14—C15—C16	−179.8 (3)
C6—C1—C7—O8	−4.1 (4)	C12—C11—C16—C15	−1.2 (4)
O7—C7—O8—C9	−4.4 (4)	C10—C11—C16—C15	179.2 (3)
C1—C7—O8—C9	175.2 (2)	C14—C15—C16—C11	0.5 (5)
C7—O8—C9—C10	−132.6 (3)		

### Hydrogen-bond geometry (Å, º)

**Table:**

*D*—H···*A*	*D*—H	H···*A*	*D*···*A*	*D*—H···*A*
C5—H5···O10^i^	0.93	2.44	3.198 (4)	139
C9—H92···O7^ii^	0.97	2.56	3.406 (4)	146

Symmetry codes: (i) −*x*, −*y*, −*z*+1; (ii) −*x*+1, −*y*+1, −*z*+1.
